# DEMENTIA PREVENTION: THE COST-EFFECTIVENESS OF PREVENTION SERVICES IN JAPANESE LONG-TERM CARE SYSTEM

**DOI:** 10.1093/geroni/igad104.3263

**Published:** 2023-12-21

**Authors:** Xujing Hu, Yunfan Wu, Miyae Yamakawa, Reiko Kanaya

**Affiliations:** Osaka University, Suita, Osaka, Japan; Osaka University, Suita, Osaka, Japan; Osaka University, Suita, Osaka, Japan; Osaka University, Suita, Osaka, Japan

## Abstract

**Objectives:**

To analyze the cost-effectiveness of prevention services (Named “Support” in Japanese official documents) in Japanese Long-Term Care Insurance system, which draws data from a large volume of 10-year database in Osaka Prefecture. Design: We design an approach to estimate cost-effective prices using aggregate data including costs of dementia in 10 years, time of dementia first diagnosed, changes of care levels, and quality of life. Measurements: The outcomes were average annual expenditure per unit of services.

**Results:**

In higher risk groups of dementia, prevention services delaying cognitive decline and enhancing quality of life were cost effective at $206 per individual. However, this cost-effectiveness will wear off as symptoms worsen and the care levels increase.

**Conclusion:**

Prevention services from Long-Term Care Insurance system are possibly to be cost-effective, but this cost benefit is not long-term .

